# Seed Nano-Priming with Calcium Oxide Maintains the Redox State by Boosting the Antioxidant Defense System in Water-Stressed Carom (*Trachyspermum ammi* L.) Plants to Confer Drought Tolerance

**DOI:** 10.3390/nano13091453

**Published:** 2023-04-24

**Authors:** Muhammad Waqas Mazhar, Muhammad Ishtiaq, Mehwish Maqbool, Syed Atiq Hussain, Ryan Casini, Ahmed M. Abd-ElGawad, Hosam O. Elansary

**Affiliations:** 1 Department of Botany, Mirpur University of Science and Technology, Mirpur 10250, Pakistan; 2 Department of Botany, University of Gujrat, Punjab 50700, Pakistan; 3 School of Public Health, University of California, Berkeley, 2121 Berkeley Way, Berkeley, CA 94704, USA; 4 Plant Production Department, College of Food and Agriculture Sciences, King Saud University, Riyadh 11451, Saudi Arabia

**Keywords:** antioxidant defense, ascorbate glutathione cycle, calcium oxide nanoparticles drought stress, *Trachyspermum ammi* L.

## Abstract

This paper explores the potential of nano seed priming with calcium oxide nanoparticles in maintaining the redox status in carom (*Trachyspermum ammi* L.) plants by modulating non-enzymatic antioxidants and enzymatic antioxidants. Calcium oxide nanoparticles were prepared in four testing regimes comprising 25, 50, 75, and 100 ppm along with the control treatment of 0 ppm (distilled water). Priming was performed by soaking the carom seeds in the aerated water, and plants were grown under split plots corresponding to drought and water. Seed priming with 75 ppm CaONPs reduced hydrogen peroxide, malondialdehyde contents and electrolyte leakage by 23.3%, 35.9% and 31.6%, respectively, in the water-stressed carom plants. The glutathione s-transferase, superoxide dismutase and peroxidase functions improved under water stress by 42.3%, 24.1% and 44.8%, respectively, in the carom plants raised through 100 ppm primed seeds with CaO_NPs. Priming induced better Ca^2+^ signaling, which affected the enzymes of the ascorbate glutathione cycle, enabling them to maintain redox status in the carom plants exposed to drought stress. The morpho-agronomic traits of carom plants in terms of number of umbels, hundred seeds weights, shoot and root length and biomass improved significantly upon seed priming treatments. Seed priming with CaO_NPs is a viable strategy to combat reactive oxygen species-mediated damages in the carom plants.

## 1. Introduction

Metabolic processes in plants lead to the formation of reactive oxygen species (ROS) as a result of the reduction of oxygen (O_2_) to water [1]. In plants, ROS is produced in various forms such as superoxide radical, singlet oxygen, hydroperoxyl radical, and hydrogen peroxide. The production of ROS is continuously countered by antioxidant defense enzymes and non-enzymatic antioxidants [2]. However, plants being sedentary in their lifestyles come across environmental stresses, which leads to a greater production of ROS, causing disturbance in the equilibrium of ROS generation and its removal. The overproduction of ROS leads to cell death due to oxidative damage, which ultimately challenges the survival of plants under harsh environmental circumstances [3]. So, there is a need to aid the antioxidant defense system of plants to effectively negotiate ROS production and counter environmental stress for their well-being and survival [4].

In plants, several enzymes are involved in defense against ROS [5]. These enzymes include superoxide dismutase (SOD), catalase (CAT), dehydroascorbate reductase (DHAR), glutathione reductase (GR), guaiacol peroxidase (GPX), and ascorbate peroxidase (APX). The enzymes DHAR, APX, and GR, are involved in ascorbate glutathione cycle (also called the Halliwell–Asada pathway). This cycle controls excessive ROS production [6]. Similarly, there are several non-enzymatic antioxidants produced within a plant to counter detrimental ROS levels. These non-enzymatic antioxidants include secondary metabolites and vitamins such as ascorbic acid and tocopherols. Plants also adjust to the sub-optimal water availability by concentrating adaptable solutes such as proline, and in the process [7], they regulate their solute composition to survive under harsh climatic conditions. Increasing the concentration of adaptable solutes and boosting antioxidant defense enables a plant to increase its yield and production under detrimental conditions [8].

Several strategies are being employed worldwide to enhance the antioxidant functions of plants, enabling them to become compatible under changing climatic events [9,10]. Seed priming or seed preconditioning is one of the promising strategies that is being employed to increase crop yield and growth. The strategy involves the controlled hydration of seeds with a pro-fertilizer before sowing [11]. By exposing the seed to stimuli, priming creates a cycle of connected biochemical changes, including the activation of enzymes, dislodging of dormancy inhibitors, and the repair of cell damage [12]. Among different forms of seed priming, nano seed priming is becoming increasingly popular with the advent of nanotechnology in agriculture. Nano seed priming is a method of seed planting preparation that involves pre-soaking the seeds in a nanoparticle solution. This novel, user-friendly, and cost-efficient approach of biofortification has increased crop protection and output in the face of deteriorating environmental circumstances that are proving detrimental to plant survival [13].

Studies have shown that stressed plants upregulate signaling cations such as Ca^2+^ and molecules such as calcium-dependent protein kinases [14,15]. These signaling molecules link plant metabolism to the changing environmental conditions, and thus, they carry out signal transduction events essential to their homeostasis. Other than signaling roles, calcium acts as an osmolyte in cellular compartments, and as a divalent cation, it concentrates soluble sugars and compatible solutes in crop species that might help plants survive under changing climates. The literature reports the ROS scavenging ability of metal oxide nanoparticles. For instance, Mazhar et al. [14] reported that nano-priming with CaO helps canola perform better under drought stress. Similarly, Nazir et al. [16] documented that CaO nanoparticles have the potential to increase the activities of antioxidant defense enzymes such as ascorbate peroxidase, superoxide dismutase and glutathione reductase.

*Trachyspermum ammi* L. (Apiaceae), commonly known as ajwain or carom plant, is a widely popular aromatic and medicinal herb. Flavone (7.1%), protein (15.4%), carbs (38.9%), moisture (8.9%), and fiber (11.9%) are all abundant in the seed of this plant [17,18]. Carom essential oil is produced from the seeds and is used extensively in folk medicine, particularly for the treatment of stomach ailments. It is also used in small amounts in food flavoring and preservation. For the treatment of asthma, a dry, hot fruit fomentation is applied externally to the chest, and a paste made of crushed fruit is administered for colic discomfort [19]. This crop is neglected in many developing nations and is mostly planted in dry, rain-fed places where there is no water available for irrigation, which causes production losses. In Pakistan, the carom crop must also contend with a hot and dry climate when the reproduction and harvesting growth stages begin [20].

A few studies report the utility of CaO_NPs in seed priming. The present work is a novel investigation since the seed priming use of CaO_NPs has not been investigated previously with reference to carom plants. Ca^2+^ signaling leads to the upregulation of associated genes and a sequence of physiological reactions such as ascorbate glutathione cycle that may empower the plants to withstand abiotic stress [20]. Our expectation is related to a nanoparticles-mediated better antioxidant defense in plants. Thus, we hypothesize that seed priming with CaO_NPs might be helpful in combating drought stress by carom plants by boosting their antioxidant defense. We expect better growth and production of carom plants raised through primed seeds. The present work aims to explore the seed priming potential of nano calcium oxide and to investigate its role as a potential seed pro-fertilizer on carom plants. The study will investigate the osmotic stress markers such as H_2_O_2_, MDA, and electrolyte leakage to access the drought induced damages. The functioning of antioxidant enzymes will be studied, and the role of a strong antioxidant defense system will be deciphered in conferring drought tolerance.

## 2. Materials and Methods

### 2.1. Priming Experiment and Experimental Layout

**Figure 1:**
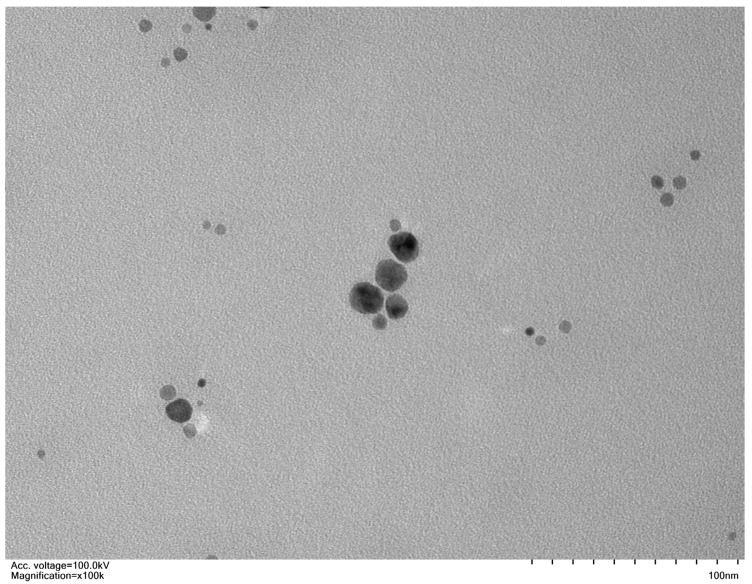
TEM image of CaO_NPs taken using FEI Tecnai 12 apparatus fitted with a digital camera from Gatan. TEM image was taken by placing CaO_NPs on a Cu grid coated with graphite.

The calcium oxide nanoparticles were obtained from Sigma-Aldrich, Steinheim, Germany, under the product number 634182. For the current experiment, nano calcium oxide whitish powder was provided. According to the manufacturers, the Brunauer–Emmett–Teller (BET) surface characterization of nano calcium oxide showed a particle size of about 160 nm. XRF (X-ray fluorescence) elemental analysis performed by the supplier states that the product nano calcium oxide contains 98% calcium metal by weight, and the XRD pattern is consistent with the structure. Figure 1 demonstrates a TEM micrograph taken to comprehend the geometry of CaO_NPs, which depicts that the supplied particles were spherical and quasi-spherical in shape. Different CaO_NPs concentrations were prepared for the seed priming treatment [14], including 0 ppm as the control treatment and trials at 25, 50, 75, and 100 ppm. CaO_NPs were subjected to ultrasonication for 30 min to ensure uniform dispersion. Carom (*Trachyspermum ammi* L.) seeds were procured from the market in good condition. After washing the seeds in 70% ethanol, the seeds were washed five times with distilled water. Priming was performed by soaking the seeds in the various concentrations of CaO_NPs. The seeds were immersed in distilled water as part of the control treatment. The priming was performed for a total of about 24 h while maintaining continuous aeration following Mazhar et al. [21].

The experimental area was divided into two equal plots corresponding to drought and well-irrigated conditions [1]. Within each plot, five subplots were created to sow the primed seeds from each of the five concentrations. Each subplot was further divided into three equal-sized rows [21]. One plant from each row was selected for data analysis, so overall, three plants (replicates) from a single seed-priming treatment were chosen for studying the physio-biochemical, yield and growth variables. Before sowing the seeds, the experimental soil was irrigated well as part of field preparation operation. At the time of field preparation, the N:P:K was administered at 60, 30, and 40 kg/ha, respectively. The experimental soil was a sandy loam, with a pH of 5.7, a total nitrogen content of 1.57%, and an EC value of 1.98 dS∙m^−1^. The soil was thoroughly tilled in preparation for seed sowing. On 1 November 2021, seeds were sown at a depth of half an inch. After 10 days of seeding, the first irrigation was applied. The schedule for reducing water stress does not include this irrigation. Following the initial irrigation for 15 days, all the plots, including those chosen for the treatment of water stress, received a second irrigation. The plants chosen for water stress treatment were only watered twice, at fully vegetative and blooming stages, while the non-stressed plots were afterwards irrigated as needed until they reached maturity. Root and shoot fresh weights were recorded at maturity; root and shoot lengths were measured. The seeds weights were recorded for one hundred seeds from the replicates, and the number of umbels was counted to record the yield attributes [22].

### 2.2. Determination of Malondialdehyde Contents

In 10 mL of TCA (10% solution made in dH_2_O), 1 g of newly collected leaf material was crushed. Then, we prepared a 0.5% TBA solution by adding 20% TCA to the supernatant (0.5 mL) of the homogenized material and incubated the mixture in a heating block at 95 °C for 50 min with constant mixing. The test tubes containing heated mixes were cooled in ice water, and subsequently, the mixes were centrifuged at 10,000× *g* for 10 min. Finally, we measured the absorbance of the colored portion at 600 nm and 532 nm to determine the MDA content [23].

MDA (nmol) = Δ (A 532 nm − A 600 nm)/1.56 × 105

The absorption coefficient for the calculation of MDA is 156 mmol^−1^ cm^−1^.

### 2.3. Hydrogen Peroxide Contents Analysis

A 0.5 g sample of fresh leaves was thoroughly mashed in 10 mL of 6% TCA. I added 1 mL of 1 M KI solution to 0.1 mL of centrifuged TCA extract. At 390 nm, the mixture’s absorbance was measured [24].

### 2.4. Determination of Electrolyte Leakage

The electrical conductivity of freshly cut leaves was evaluated after dipping them in a test tube containing 10 mL of double-distilled water (EC0). In a temperature-controlled dry block heater, the sample tubes were heated to boiling temperatures of 50 °C and 100 °C for 20 and 10 min, respectively, before the corresponding electrical conductivities (EC1 and EC2) were simultaneously measured [25].

EC (%) = (EC1 − EC0)/(EC2 − EC0) × 100(1)

### 2.5. Determination of Oxidized and Reduced States of Glutathione

The glutathione contents in leaves were measured. The procedure for analyzing fresh leaf material for its glutathione contents involved the following steps: First, 250 mg of fresh leaf material of carom was ground in 0.1 M HCl (2 mL) containing EDTA at a concentration of 1 mM. Next, the extract was collected by centrifuging the mixture at 12,000× *g* for 15 min at a temperature of 4 °C. A reaction mixture was prepared by adding 200 mL of phosphate buffer (125 mM), 100 mL of DTNB (6.0 mM), 200 mL of the extract, and 500 mL of NADPH, resulting in a concentration of 0.3 mM. Finally, the absorbance of the reaction mixture was measured at 412 nm. [26].

### 2.6. Shoot and Root Calcium Contents

Sulfuric acid was used to digest powdered materials, as suggested by Wolf [27]. Atomic Ab. Spectro Model 7JO-8024 was used to analyze the contents of root and shoot Ca^2+^.

### 2.7. Determination of Tocopherols

To determine the tocopherol content in fresh leaf material, the following steps were taken: First, 0.5 g of leaf material from each sample was centrifuged at 10,000× *g* for 20 min and mixed with 10 mL of a 2:1.6 (*v*/*v*) mixture of petroleum ether and ethanol. An aliquot of 1 mL was taken and added to 200 L of 2-dipyridyl in ethanol (2%), and the mixture was agitated before being left in the dark for five minutes. Afterward, 4 mL of distilled and deionized water was added to the mixture and carefully stirred. The tocopherol content was determined by measuring the amount of α-tocopherol, which was used to create a standard curve for calculation. The measurement was made at 520 nm using a spectrophotometer [28].

### 2.8. Determination of Superoxide Dismutase (SOD) and Peroxidase (POD) Activity

Based on the photochemical reduction inhibition principle of nitro blue tetrazolium (NBT) in light, SOD activity was evaluated. At 560 nm, the decrease inhibition of NBT was detected. In brief, the study’s produced combination contained 50 mM phosphate buffer, 75 nM EDTA, 50 mM NBT, 1.3 mM riboflavin, and 13 mM methionine (pH 7.8). The interior aluminum foil coating in a box with fluorescent lighting (20 W) for 15 min. The reaction mixture’s absorbance was then measured at 560 nm with a UV-visible spectrophotometer [29,30].

POD activity was assessed based on the oxidation of guaiacol present in the reaction mix. As a measure of POD activity, 0.01 absorbance units per minute per milligram of protein was used [30].

### 2.9. Determination of Glutathione S-Transferase Activity and Ascorbate Glutathione Pathway Enzymes

To determine the activity of glutathione S-transferase, the method described by Hasanuzzaman and Fujita [31] was used. The reaction mixture was prepared by adding 100 mM Tris-HCl buffer (pH 7.0), 1 mM GSH, 1 mM 1-chloro-2,4-dinitrobenzene (CDNB), and enzyme extract. The reaction was initiated, and the absorbance of the mixture was measured at 340 nm using a spectrophotometer (Beckman 640 D, Brea, CA, USA).

Nakano and Asada’s method [32] was used to measure the ascorbate peroxidase activity. Using a spectrophotometer, the H_2_O_2_-based oxidation of ascorbate was measured at 290 nm for two minutes.

The quantification of glutathione reductase activity was carried out using the Foster and Hess method [33]. The reaction mixture contained enzyme extract, 500 mM GSSG, 150 mM NADPH, 1 mM EDTA, and 100 mM potassium phosphate buffer (pH 7.0). The varying absorbance color intensity was measured at 340 nm using a spectrophotometer (Beckman 640D, Brea, CA, USA) for three minutes. The activity of monodehydroascorbate reductase was measured using the Miyake and Asada method [34], and it was expressed as MDHAR activity in mol NADPH oxidized (EU mg1 protein). The activity of dehydroascorbate reductase (DHAR, 1.8.5.1) was measured according to the recommended procedure [32]. The absorbance was measured at 265 nm using a spectrophotometer (Beckman 640D, Brea, CA, USA).

### 2.10. Determination of Ascorbic Acid (AsA) Contents

To prepare the sample, 0.25 g of fresh leaf was finely ground and mixed with 10 mL of a 6% trichloroacetic acid solution. After centrifugation at 10,000× *g* for 10 min, the supernatant was treated with a 2% acidic dinitrophenyl hydrazine solution (2 mL). A drop of 10% thiourea was added to the resulting mixture, which was then heated in a water bath for 20 min. After the liquid had been cooled to 0 °C, 80% H_2_SO_4_ (5 mL) was added. At 530 nm, the OD of the finished colored substance was measured. Pure AsA was used to calculate the concentration in the leaf sample samples, and a range of standard solutions (50–300 ppm) were used to create the standard curve [35].

### 2.11. Statistical Analysis

Statistical analysis in terms of least significance difference (LSD) test and analysis of variance (ANOVA) was performed using Costat software version 6.3, which was developed by Cohort software Berkeley USA. For principal component analysis (PCA) and Spearman correlation matrix, XLSTAT was employed.

## 3. Results

**Figure 2:**
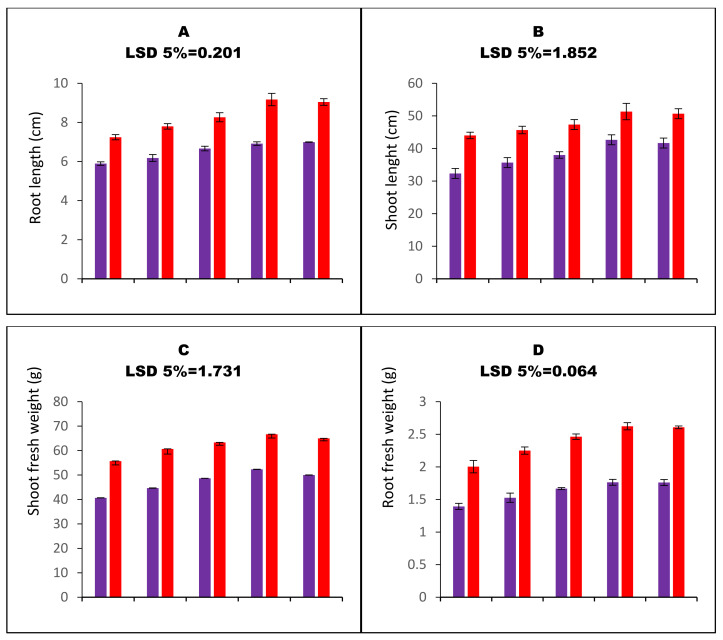

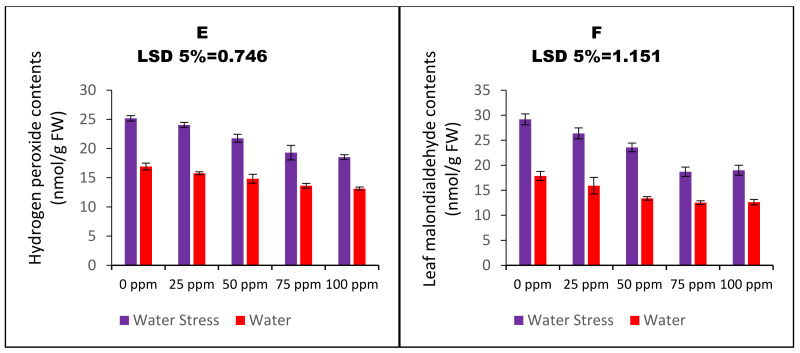
Bar charts (mean ± S.E; n = 3) representing growth attributes and activities of assayed non-enzymatic antioxidants of carom plants (*Trachyspermum ammi* L.) raised from calcium oxide nanoparticles primed seeds grown under water stress. 0, 25, 50, 75, and 100 ppm on the x-axis represent concentrations of CaO-NPs followed in the seed priming experiment.

All the studies’ growth parameters were decreased upon the imposition of water stress. Water stress decreased the root length, shoot length, shoot fresh weights, and root fresh weights of carom plants by 18.6%, 26.5%, 26.9%, and 30.4%, respectively, compared to respective control treatment. However, the seed priming (SP) resulted in better growth and biomass characteristics of carom plants. The 75 ppm priming concentration enhanced the root length, shoot length, shoot fresh weights, and root fresh weights of water-stressed carom plants by 17.3%, 31.9%, 28.6%, and 26.5%, respectively (Figure 2A–D).

**Figure 3:**
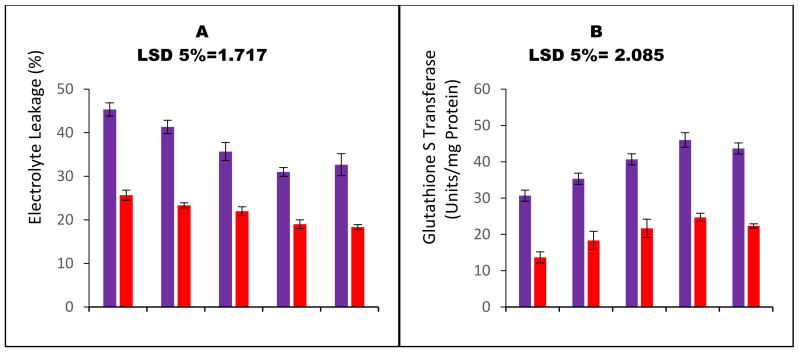

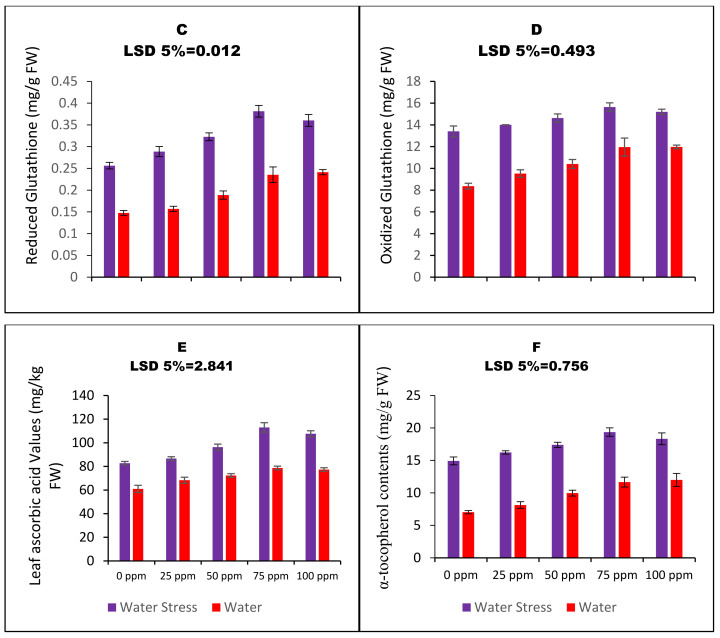
Bar charts (Mean ± S.E; n = 3) representing elevated concentrations of osmotic stress markers and glutathione in carom plants (*Trachyspermum ammi* L.) raised from calcium oxide nanoparticles primed seeds grown under water stress. 0, 25, 50, 75, and 100 ppm on x-axis represent concentrations of CaO-NPs followed in the seed priming experiment.

Water stress increased hydrogen peroxide contents by 32.7% compared to the well-irrigated plots. All the SP application significantly decreased the hydrogen peroxide contents (Figure 2E). SP with 75 ppm CaO_NPs decreased the contents of hydrogen peroxide in water-stressed carom plants by 23.3%. Similarly, the levels of the lipid peroxidation product, malondialdehyde (MDA), were significantly enhanced under the drought stress. Water-stressed carom plants accumulated MDA 38.7% more compared to the well-irrigated plants (Figure 2F). SP with CaO_NPs significantly decreased the contents of MDA. A 35.9% decrease in the MDA accumulation was observed in the plants raised from 75 ppm CaO_NPs. Furthermore, the carom plants raised under water stress showed 43.3% more electrolyte leakage, indicating the amount of tissue damage upon the imposition of water stress (Figure 3C). However, the carom plants raised through CaO_NPs primed seeds showed significantly lower tissue damage. SP with 75 ppm CaO_NPs reduced electrolyte leakage by 31.6% under drought stress and proved the best among all the treatments. All the assayed stress markers were found significantly reduced upon SP in the well-irrigated plots as well.

When carom plants were exposed to water stress, there was a noticeable rise in GST activity (Figure 3B). Additionally, the levels of glutathione’s redox forms were measured and found to be significantly higher under drought stress conditions. The application of CaO_NPs during SP further enhanced the glutathione levels and resulted in a significant increase in GST activity under both irrigated and water-stressed conditions. SP with 100 ppm CaO_NPs proved most beneficial in elevating the activity of GST and glutathione as the treatment boosted GST activity by 42.3%, and it further enhanced reduced and oxidized glutathione contents by 40.5% and 13.3%, respectively (Figure 3C,D).

Water stress resulted in the elevated vitamin antioxidants status of carom plans. The activities of α-tocopherol and ascorbic acid were assayed as a part of the experimental plan. The carom plants raised through 75 ppm CaO_NPs primed seeds experienced enhanced ascorbic acid contents by 22% and that of α-tocopherol by 26%. The effect of SP was significant under both water-stressed and well-irrigated plots (Figure 3E,F).

**Figure 4:**
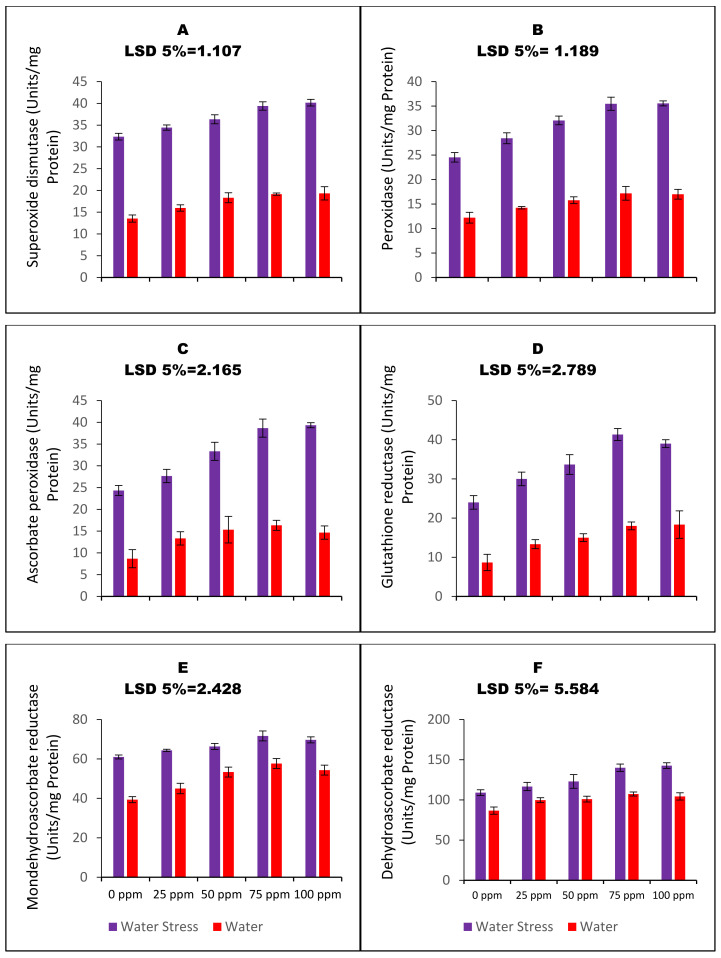
Bar charts (mean ± S.E; n = 3) representing the functioning of antioxidant enzymes and enzymes of the ascorbate–glutathione pathway conferring the induction of drought stress tolerance in carom plants (*Trachyspermum ammi* L.) raised from calcium oxide nanoparticles primed seeds. 0, 25, 50, 75, and 100 ppm on the x-axis represent concentrations of CaO_NPs followed in the seed priming experiment.

The carom plants subjected to drought stress exhibited a significant increase in the activity of SOD and POD enzymes (Figure 4A,B). The use of CaO_NPs for SP further enhanced the function of these enzymes with a concentration of 100 ppm showing the most effective results. The treatment resulted in 24.1% and 44.8% increases in the activity of SOD and POD, respectively, in carom plants grown under drought stress. Additionally, drought stress increased the activity of other enzymes involved in the ascorbate–glutathione cycle, including ascorbate peroxidase (Figure 4C), glutathione reductase (Figure 4D), monodehydroascorbate reductase (Figure 4E), and dehydroascorbate reductase (Figure 4F). SP with CaO_NPs further upregulated the functioning of these enzymes, with all priming regimes significantly increasing the activity of these enzymes under both irrigation regimes. Specifically, the plants primed with 100 ppm CaO_NPs showed a further 61.6% increase in ascorbate peroxidase activity, a further 62.5% increase in glutathione reductase activity, a further 14.2% increase in monodehydroascorbate reductase activity, and a further 30.8% increase in dehydroascorbate reductase activity under drought stress.

**Figure 5:**
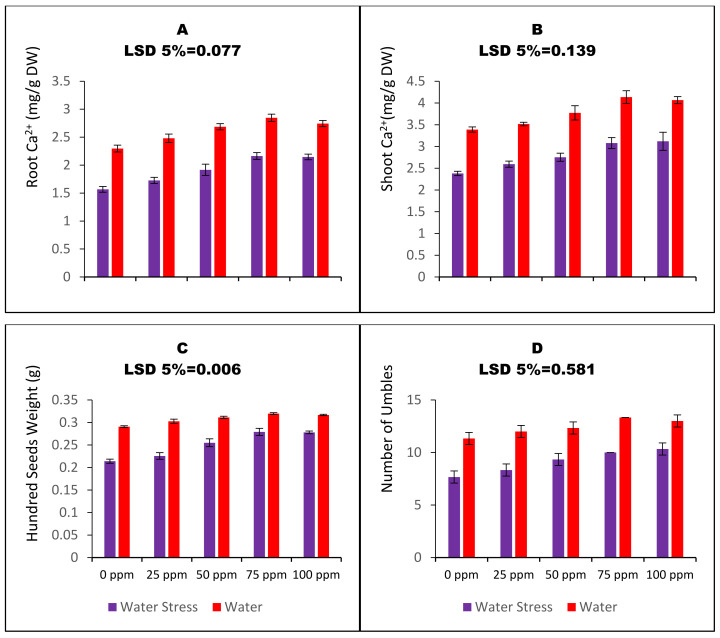
Bar charts (mean ± S.E; n = 3) representing root and shoot Ca^2+^ and recorded yield attributes of carom plants (*Trachyspermum ammi* L.) raised from calcium oxide nanoparticles primed seeds grown under water stress. 0, 25, 50, 75, and 100 ppm on the x-axis represent concentrations of CaO_NPs followed in the seed priming experiment.

Water-stressed carom plants were poor in uptake of Ca^2+^, as lower water availability resulted in reduced root and shoot Ca^2+^ acquisition, i.e., by 31.7% and 29.7%, respectively (Figure 5A,B). The plants raised through CaO_NPs primed seeds were better in Ca^2+^ contents under both irrigation regimes. SP with 75 ppm CaO_NPs increased root and shoot Ca^2+^ contents by 38% and 29.4%, respectively, in the water-stressed carom plants. Likewise, the plants raised through CaO_NPs-mediated SP were better in measured yield parameters such as the number of umbels and seed weights. Water stress resulted in a 32% decrease in the number of carom umbels and 26.3% decrease in hundred seeds weight. SP with a 75 ppm concentration of nano calcium oxide increased the number of umbels and hundred seeds weights of water-stressed carom plants by 30.4% and 30.3%, respectively.

**Figure 6:**
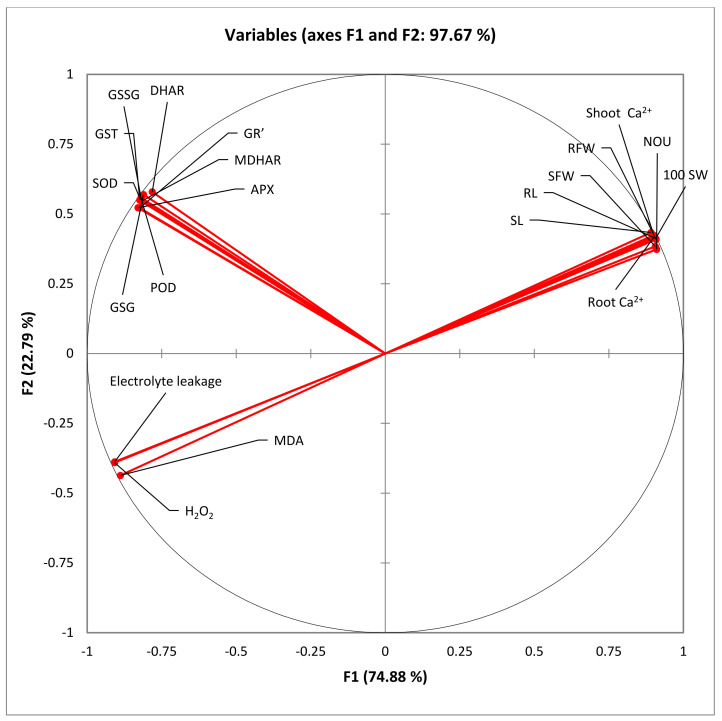
Principal component analysis (PCA) of various observed parameters followed in the study. H_2_O_2_: hydrogen peroxide; RFW: root fresh weights; SFW: shoot fresh weights; NOU: number of umbels; RL: root length; SL: shoot length; DHAR: dehydroascorbate reductase; GST: glutathione s-transferase; APX: ascorbate peroxidase; DR: glutathione reductase; POD: peroxidase; MDA: malondialdehyde; SOD: superoxide dismutase; GSG: reduced glutathione; GSSG: oxidized glutathione; 100 SW: hundred seeds weights; MDHAR: monodehydroascorbate reductase.

The PCA loading plot as presented in Figure 6 clearly demonstrates that all the studied variables of carom plants were significantly correlated in mitigating drought stress. The circle diagram depicts that the root length, shoot length, yield parameters and root and shoot calcium levels are all in one quarter, as all of these variables are decreased under water stress and are improved if the carom plants are raised through CaO_NPs primed seeds. All the antioxidant enzymes and the enzymes of ascorbate glutathione cycle (AsA-GSH cycle) can be seen on the other quarter, as all of these are upregulated under drought stress as well as by SP. However, the osmotic stress markers such as MDA, H_2_O_2_, and electrolyte leakage all lie in a different quarter, as these variables were increased under water stress, but their contents decreased upon the SP treatments.

Spearman correlation matrix was constructed to comprehend the correlation among the variables. Table 1 shows that all of the variables are either positively or negatively yet significantly correlated. In Table 2, we have presented the results from two-way analysis of variance in terms of *p*-value and mean square. The results are significant on various *p* values at significance levels alpha 0.05, 0.01, and 0.001.

## 4. Discussion

Recent research momentum has resulted in the discovery of a number of Ca^2+^ binding transcription factors that may serve as signaling candidates to help the plant cope with environmental stresses [36]. Therefore, we exploited the CaO_NPs as seed preconditioners in reducing drought stress in the carom plants. Shoot and root Ca^2+^ contents were improved significantly under seed treatment with CaO_NPs. The increased levels of Ca^2+^ result in the synthesis of polyamines, which act as growth stimulators and improve seed germination. Furthermore, Ca^2+^ regulates transcriptional networks that may lead to the production of amylase for food mobilization and seed germination because on-time transcription of the appropriate genes is the fundamental mechanism in regulating healthy growth in plants [37]. Therefore, the application of CaO_NPs might be responsible for a better drought-tolerant mechanism in carom plants.

The functioning of plant defense enzymes upregulated under the drought also changed. Seed priming with CaO_NPs further increased the activities of SOD and CAT. SOD performs the dismutation of superoxide radical to hydrogen peroxide, which is reduced directly by the functioning of ascorbate peroxidase (APX) into water. Ca^2+^ modulates genes that protect plants against environmental hazards [38]. Presumably, CaO_NPs primed seeds might have upregulated the genes responsible for initiating a stress tolerance response in carom plants that ultimately boosted SOD and CAT activities, leading to a reduction in ROS levels. Intracellular Ca^2+^ fluctuation is one of the first things to occur after a plant recognizes an external stimulus. For decoding and relaying them into biological reactions, Ca^2+^ sensors are required. Calmodulin (CaM) sensors indicate the presence of specialized machinery and tools in plants that allow them to translate Ca^2+^ signals into the appropriate reactions [39]. It is possible to conclude that seed priming could perhaps boost carom plants’ resistance to drought due to role of calcium in signaling.

In the current investigation, we found that water shortage conditions boosted hydrogen peroxide production, and an increased electrolyte leakage was examined in response to the imposition of drought stress. Similarly, an increased lipid peroxidation product malondialdehyde (MDA) was assayed upon drought treatment to the carom plants. The levels of these stress markers were dramatically reduced after CaO_NPs primed seeds. The increased antioxidant enzyme activity brought on by CaO_NPs causes a decrease in H_2_O_2_ [40,41]. The breakdown of membrane lipids results in the accumulation of MDA. Calcium has the capacity to bind to membranes, stabilizing the membrane structure as a result [42]. Presumably, CaO_NPs primed seeds might have a stabilized membrane structure in the carom plants, lowering MDA contents and electrolyte leakage. Furthermore, calcium preserves Hill’s reaction while reducing NADPH oxidase activity to reduce MDA, EC, and H_2_O_2_. Under fragile environments, CaO_NPs primed seeds might serve as a promising tool to enhance crop performance [43].

All the studied growth parameters were decreased upon the imposition of water stress. The growth characteristics considerably enhanced after CaO_NPs mediated the seed priming of carom plants. In this respect, Sarkar et al. [44] reported that seed priming leads to the activation of genes which express to produce the α-amylase enzyme. The enzyme is involved in catalyzing the hydrolysis of starch into soluble sugars. Presumably, the build-up of soluble sugar from seed priming treatments may be the cause of better growth parameters of carom plants. Soluble sugars are transferred from seeds to multiple seedling organs during the germination process [45]. Presumably, a better metabolism of sugars might have resulted in a better seed germination response, and thus, this overall process reflected a better growth of carom plants. Similar results were reported by Hosseini et al. [46], which state that calcium treatment increases the synthesis of plant biomass by stimulating the accumulation of sugars and osmolytes in the leaves and the uptake of Mg and Si. Additionally, the role of Ca^2+^ in stabilizing the plastids’ structural envelope may be the cause of the rise in biomass [47]. This stabilization of plastid membrane was evident, as seed priming reduced MDA levels in the leaves of carom plants as reported by our results.

Growing the carom plants from primed seeds with CaO_NPs led to an improvement in the yield characteristics observed in terms of the number of umbels and seed weights. Increased crop productivity and improved agronomic qualities may be caused by nutrient treatment with CaO_NPs, which also improves soil fertility [48]. In addition to being a nutrient in and of itself, calcium also serves as a signaling molecule for the uptake of other minerals by plants, including potassium, nitrate, and ammonium [49]. Furthermore, nano-priming leads to the expression of genes involved in better water use efficiency. Presumably, due to the expression of the aquaporin gene being driven by nanoparticles, carom plants behaved better in terms of yield and were therefore better able to withstand drought conditions [14,50].

Drought stress significantly raised endogenous ascorbic acid. CaO_NPs primed seeds further raised the levels of ascorbic acid. The ascorbic acid is a water-soluble compound which forms ascorbate anion. Both forms are interconvertible [51]. Ascorbate is abundant and essential for resistance to oxidative stress brought on by high levels of ROS: it is a key component. The Smirnoff–Wheeler pathway, which uses d-mannose/l-galactose, produces the AsA pool in plants [52]. Seed priming leads to the activation of genes which express to produce the α-amylase enzyme. Presumably, a better sugar metabolism upon seed priming might have upregulated the Smirnoff–Wheeler pathway, leading to a greater accumulation of ascorbate. In plants, AsA is taken to represent the initial line of defense against potentially harmful exogenous oxidants [53].

CaO_NPs primed seeds increased the glutathione pool in the water-stressed carom plants. Glutathione (GSH) makes a considerable contribution to intracellular protection against ROS. [54]. With its reducing power, GSH contributes significantly to a number of biological acts, including cell division and growth, sulfate regulation, signal transduction, metabolite conjugation, enzymatic control, the synthesis of proteins and nucleic acids, phytochelatin synthesis, formatal chelation, and xenobiotic detoxification [55]. Due to its ability to chemically interact with ROS, GSH serves directly as a free radical scavenger. As GSH forms adducts with reactive electrophiles, GSSG is created, protecting the macromolecules. During the AsA-GSH cycle, it also aids in the regeneration of ascorbate [56]. The conjugation of glutathione (GSH) to a wide range of endogenous and exogenous electrophilic chemicals is catalyzed by the GSTs family of phase II detoxification enzymes. In the present study, we report the increased functioning of GST upon CaO_NPs-mediated seed priming of carom plants. These results are in accordance with [57].

Primed seeds slow the release of fertilizers such as priming chemicals from the seed coats, allowing the plant to tolerate oxidative stress by raising the concentration of antioxidant enzymes [58]. A more robust antioxidant defense enables the plant to withstand ROS more successfully, assisting in the fight against disease and fostering growth in challenging climatic conditions [59]. In the current study, we report an increased functioning of the enzymes involved in the AsA–GSH cycle upon seed priming with CaO_NPs. These enzymes include APX, GR, MDHAR, and DHAR. In this respect, Jing et al. [60] reported that calcium increases the activity of antioxidant enzymes. The enzyme APX is involved in the AsA–GSH cycle, and furthermore, the detoxification of hydrogen peroxide is performed by the enzymes of this cycle. APX uses ascorbate for this reaction, which is produced by the activity of dehydroascorbate reductase (DHAR). The ascorbate is produced by the action of enzyme of glutathione reductase (GR), which utilizes reduced glutathione for this purpose. Presumably, a better antioxidant defense system might have enabled the carom plants to perform better under drought stress. Calcium signaling influences the jasmone acid-mediated signaling system inside plant cells via calcium channels. Shan et al. [61] reported that jasmonic acid biosynthesis leads to the production of nitric oxide which regulates the AsA–GSH cycle under abiotic stress. From these findings, presumably, Ca supplementation through nano-priming might have enabled carom plants to confer a drought tolerance response due to the effective mitigation of hydrogen peroxide through the AsA–GSH cycle. Furthermore, during the ascorbate–glutathione (AsA–GSH) cycle, AsA is crucial for the removal of H_2_O_2_. With the aid of two molecules of AsA, APX converts H_2_O_2_ to water while also producing monodehydroascorbate (MDHA). The MDHAR enzyme, which is dependent on NADP(H), directly reduces the short-lived MDHA radical to AsA or causes it to spontaneously dismutase into dehydroascorbate (DHA) and AsA, increasing the ascorbate pool. The previous studies reported by Ahmad et al. [62] have shown that calcium supplementation causes the antioxidant system in mustard to be upregulated as reported by our results. Drought stress increased the levels of assayed α-tocopherol contents, which were further boosted upon seed priming with CaO_NPs. Tocopherols preserve lipids and other membrane elements, particularly PSII, by physically quenching and chemically reacting with O_2_ in chloroplasts. Before it degrades, a single molecule of α-tocopherol can neutralize a significant amount of O_2_ molecules. The conversion of oxidized tocopherol to its reduced form is aided by AsA and GSH. Presumably, CaO_NPs primed seeds boosted the enzymes of the AsA–GSH cycle, which increased α-tocopherol contents [63].

From the above discussion, it can be concluded that Ca aids in the perception of stress signals by membrane receptors, the production of secondary messengers, the targeting of transcription factors and the regulation of gene expression that leads to the development of stress tolerance in carom plants [14]. Apart from numerous applications of nanotechnology in agriculture such as nano-seed priming, nano sensors and nano clays, the nanomaterials are finding a huge spectrum of applications in other fields of life science. For instance, in a study [64], the authors utilized gold nanoprobes as potential applications for understanding gene expression patterns and regulation in plants as well as for studying the dynamics of rare cell types or cell states in complex tissues.

## 5. Conclusions

The aim of this study was to investigate whether seed priming with CaO_NPs could enhance the antioxidant defense system of carom plants and provide drought tolerance. The results of the experiment were as expected, as seed priming with CaO_NPs improved the growth and production of carom plants under drought stress in split plot trials. In water-stressed carom plants, seed priming with 75 ppm of CaO nanoparticles significantly reduced levels of hydrogen peroxide, malondialdehyde, and electrolyte leakage by 23.3%, 35.9%, and 31.6%, respectively. Seed priming with CaO_NPs increased the accumulation of vitamin antioxidants and improved the antioxidant defense of carom plants by upregulating the functioning of superoxide dismutase and peroxidase. Furthermore, the functioning of four enzymes involved in the AsA–GSH cycle was improved by seed priming treatments. The results suggest that seed priming with CaO_NPs may be a useful approach to improve the growth and production of carom plants in arid regions. The authors suggest further exploration in the area and further explorations in terms of other nanomaterials and the mode of their application such as foliar sprays and fertigation. The usability of CaO_NPs can be applied through different methods such as foliar spray, soil application, or seed coating in future plant-related applications.

## Figures and Tables

**Table 1:** Spearman correlation matrix showing significant correlation among various physiochemical parameters of carom plants (*Trachyspermum ammi* L.) plants raised through calcium oxide nanoparticles primed seeds.

Variables	^a^ H_2_O_2_ Values	MDA Contents	E Leakage	SOD Activity	POD Activity	GST Activity	APX Activity	GR’ Activity	MDHAR Activity	DHAR activity	GSG Activity	GSSG Activity	Root Ca^2+^	Shoot Ca^2+^	100 SW
H_2_O_2_	1														
MDA	0.977 *	1													
E Leakage	0.977 *	0.978 *	1												
SOD	0.521 *	0.494 *	0.536 *	1											
POD	0.515 *	0.488 *	0.532 *	0.988 *	1										
GST	0.546 *	0.492 *	0.538 *	0.976 *	0.972 *	1									
APX	0.550 *	0.518 *	0.564 *	0.977 *	0.980 *	0.969 *	1								
GR	0.553 *	0.490 *	0.527 *	0.944 *	0.952 *	0.973 *	0.933 *	1							
MDHAR	0.538 *	0.476 *	0.534 *	0.975 *	0.965 *	0.984 *	0.965 *	0.963 *	1						
DHAR	0.479 *	0.458 *	0.488 *	0.969 *	0.965 *	0.956 *	0.950 *	0.924 *	0.935 *	1					
GSG	0.511 *	0.472 *	0.511 *	0.974 *	0.985 *	0.973 *	0.958 *	0.970 *	0.965 *	0.961 *	1				
GSSG	0.527 *	0.481 *	0.520 *	0.970 *	0.980 *	0.976 *	0.953 *	0.980 *	0.968 *	0.952 *	0.995 *	1			
Root Ca^2+^	−0.975 *	−0.974 *	−0.978 *	−0.511 *	−0.507 *	−0.508 *	−0.521 *	−0.531 *	−0.514 *	−0.460 *	−0.496 *	−0.511 *	1		
Shoot Ca^2+^	−0.985 *	−0.984 *	−0.972 *	−0.499 *	−0.496 *	−0.510 *	−0.520 *	−0.535 *	−0.509 *	−0.453 *	−0.491 *	−0.507 *	0.989 *	1	
100 SW	−0.978 *	−0.990 *	−0.980 *	−0.518 *	−0.521 *	−0.516 *	−0.538 *	−0.526 *	−0.508 *	−0.472 *	−0.507 *	−0.519 *	0.988 *	0.990 *	1
NOU	−0.971 *	−0.968 *	−0.956 *	−0.540 *	−0.538 *	−0.540 *	−0.552 *	−0.560 *	−0.543 *	−0.499 *	−0.529 *	−0.537 *	0.972 *	0.980 *	0.975 *

^a^ H_2_O_2_: hydrogen peroxide; NOU: number of umbels; MDHAR: monodehydroascorbate reductase; DHAR: dehydroascorbate reductase; GST: glutathione S-transferase; APX: ascorbate peroxidase; DR: glutathione reductase; POD: peroxidase; MDA: malondialdehyde; SOD: superoxide dismutase; GSG: reduced glutathione; GSSG: oxidized glutathione; 100 SW: hundred seeds weights. Values with * are different from 0 with a significance level alpha = 0.05.

**Table 2:** Physiochemical parameters of carom plants (*Trachyspermum ammi* L.) measured after seed priming using calcium oxide nanoparticles and analyzed through two-way ANOVA (with mean square and *p*-values).

Variation Source	^a^ *df*	100 SW	NOU	RL	SL	RFW	Ascorbic Acid	α-Tocopherols
Water Stress (WS)	1	0.025 ^b,^*** (0.000)	80.033 *** (0.000)	23.603 *** (0.000)	710.533 *** (0.000)	4.416 *** (0.000)	4966.345 *** (0.000)	421.12 *** (0.000)
CaONPs Seed Priming (SP)	4	0.002 *** (0.000)	5.383 *** (0.000)	2.490 *** (0.000)	82.383 *** (0.000)	0.267 *** (0.000)	602.8 *** (0.000)	22.418 *** (0.000)
WS X SP	4	0.001 *** (0.000)	0.283 ns (0.335)	0.202 *** (0.000)	2.116 ns (0.451)	0.015 ** (0.003)	63.553 *** (0.000)	0.722 ns (0.162)
Error	20	0.00	0.233	0.028	2.366	0.002	5.566	0.394
**Variation Source**	***df***	**Electrolyte Leakage**	**H_2_O_2_**	**MDA**	**SOD**	**POD**	**GST**	**APX**
Water Stress (WS)	1	1809.633 *** (0.000)	357.213 *** (0.000)	594.075 *** (0.000)	2788.633 *** (0.000)	1904.192 *** (0.000)	2745.63 *** (0.000)	2707.5 *** (0.000)
CaONPs Seed Priming (SP)	4	121.05 *** (0.000)	29.275 *** (0.000)	70.365 *** (0.000)	48.783 *** (0.000)	69.852 *** (0.000)	164.616 *** (0.000)	129.58 *** (0.000)
WS X SP	4	15.216 *** (0.000)	2.824 *** (0.000)	8.878 *** (0.000)	2.164 ns (0.070)	10.733 *** (0.000)	7.05 ns (0.089)	28.916 *** ns (0.000)
Error	20	2.033	0.384	0.914	0.846	0.974	3	0.001
**Variation Source**	***df***	**GR**	**MDHAR**	**DHAR**	**GSG**	**GSSG**	**Root Calcium**	**Shoot Calcium**
Water Stress (WS)	1	2688.533 *** (0.000)	2083.333 *** (0.000)	5253.633 *** (0.000)	5096.33 *** (0.000)	127.965 *** (0.000)	3.745 *** (0.000)	7.370 *** (0.000)
CaONPs Seed Priming (SP)	4	177.2 *** (0.000)	203.883 *** (0.000)	717.616 *** (0.000)	492.283 *** (0.000)	9.015 *** (0.000)	0.342 ***(0.000)	0.619 ***(0.000)
WS X SP	4	15.2 ns (0.051)	20.416 ** (0.005)	115.716 ** (0.004)	67.783 *** (0.000)	0.741 * (0.010)	0.007 ns (0.181)	0.004 ns (0.8512)
Error	20	5.36	4.066	2.528	6.133	0.168	0.004	0.001

^a^ *df*: degree of freedom, ns non-significant, H_2_O_2_: hydrogen peroxide; NOU: number of umbels; MDHAR: monodehydroascorbate reductase; DHAR: dehydroascorbate reductase; GST: glutathione S transferase; APX: ascorbate peroxidase; DR: glutathione reductase; POD: peroxidase; MDA: malondialdehyde; SOD: superoxide dismutase; GSG: reduced glutathione; GSSG: oxidized glutathione; 100SW: hundred seeds weights; RL: root length; SL: shoot length; RFW: root fresh weight. ^b^ *, **, and *** = significant at 0.05, 0.01, and 0.001 levels, respectively.

## Data Availability

All data relevant to this study are included in the manuscript.
